# Modulation of Iron Metabolism in Response to Infection: Twists for All Tastes

**DOI:** 10.3390/ph11030084

**Published:** 2018-09-01

**Authors:** Ana Cordeiro Gomes, Ana C. Moreira, Gonçalo Mesquita, Maria Salomé Gomes

**Affiliations:** 1 Instituto de Investigação e Inovação em Saúde, Universidade do Porto, 4200-135 Porto, Portugal; ana.c.gomes@i3s.up.pt (A.C.G.); ana.s.moreira@ibmc.up.pt (A.C.M.); goncalo.mesquita@i3s.up.pt (G.M.); 2 Instituto de Biologia Molecular e Celular, Universidade do Porto, 4200-135 Porto, Portugal; 3 Instituto de Ciências Biomédicas de Abel Salazar, Universidade do Porto, 4050-313 Porto, Portugal

**Keywords:** iron metabolism, infection, innate immunity, hepcidin, ferritin, anemia of inflammation, pharmaceutical targets

## Abstract

Iron is an essential nutrient for almost all living organisms, but is not easily made available. Hosts and pathogens engage in a fight for the metal during an infection, leading to major alterations in the host’s iron metabolism. Important pathological consequences can emerge from the mentioned interaction, including anemia. Several recent reports have highlighted the alterations in iron metabolism caused by different types of infection, and several possible therapeutic strategies emerge, based on the targeting of the host’s iron metabolism. Here, we review the most recent literature on iron metabolism alterations that are induced by infection, the consequent development of anemia, and the potential therapeutic approaches to modulate iron metabolism in order to correct iron-related pathologies and control the ongoing infection.

## 1. Introduction

The virulence of a pathogen is directly related to its capacity to adapt to the environment present within the host, and also its ability to escape or subvert the host’s immune response. Amongst all the nutritional needs of the pathogens, the acquisition of iron is probably one of the major determinants for their maintenance and proliferation within a host. Most pathogens synthesize small molecules, called siderophores, which have a high affinity for iron, to ensure their iron acquisition. There are several types of siderophores and the same bacterial species can produce different molecules [1]. Several pathogens also have heme uptake systems and are able to take up iron from transferrin or other host iron-binding proteins [2]. Additional evidence for the importance of iron, during an infection, comes from epidemiological studies which correlate between the host’s iron status and the clinical outcome of several infections [3]. For instance, the iron status of the host, at the time of an HIV infection diagnosis, modulates the risk for subsequent development of tuberculosis [4]. Additionally, iron-supplementation programs increase the risk of malaria, diarrhea and respiratory infections in endemic regions [5,6]. It is increasingly recognized that together with the other components of the immune response, the host uses a variety of mechanisms and strategies to deprive pathogens of the essential nutrients, such as iron, zinc, and copper [7]. This host response to infection has been coined “nutriprive” or “nutritional immunity” and results from extensive fine-tuning throughout animal evolution [8,9]. Nutritional immunity, and in particular iron deprivation, may be a valuable target in the development of host-directed therapies against infection. However, this development would require a deeper knowledge of the iron metabolic and distribution pathways, which are essential not only for pathogens but also for the host.

Animals are highly dependent on iron for fundamental processes such as DNA replication, oxygen transport, and immune function. Conversely, iron’s high reactivity makes it highly toxic when left in a free form. Therefore, iron acquisition, storage, and transport are tightly regulated processes [10]. An increasing amount of data shows that infections have an enormous impact on the host’s iron metabolism and distribution, not only through the innate mechanisms of iron withholding but also by the effects of the pathogen, the immune response, tissue damage or other indirect consequences of an infection. The diverse effects seen in different infections may have host-protective as well as host-deleterious consequences, the most well-known of which is anemia of infection.

Here, we will review the most recent evidence linking iron metabolism and infection, highlighting studies that have been performed in vivo, which put in an evidence for systemic rather than cell-intrinsic alterations. We will also address the mechanisms leading to anemia of inflammation, which is an important co-morbidity associated with infections and iron availability. Finally, we will discuss the potential modulation of these pathways to be used as therapeutic targets in the clinics.

## 2. Iron Homeostasis in Animal Hosts

Due to its high toxicity, the amount of iron in the body must be tightly regulated. There are no known active iron excretion systems in mammals. Therefore, only small amounts of iron are normally lost by bleeding, the sloughing of mucosal cells, the desquamation of skin cells, and in the urine. Conversely, iron uptake from the diet is tightly regulated. In mammals, iron is absorbed mostly in the proximal duodenum, either in the ferrous form, through the divalent metal transporter 1 (DMT-1) or in the form of heme, presumably through the heme carrier protein 1 (HCP-1). However, the identity of the transporter(s) involved is still a matter of debate [11,12]. Once inside the enterocytes, heme is degraded by the enzyme heme oxygenase 1 (HO-1) into carbon monoxide, bilirubin, and iron. Absorbed iron can be used by the body, stored intracellularly in ferritins, or exported through the ferroportin 1 (FPN1) and complexed with the transferrin in the serum. Most of the circulating iron reaches all tissues being safely bound to transferrin, and most cells acquire iron through the receptor-mediated endocytosis of iron-bound transferrin. However, in situations of iron overload, the amount of iron in circulation may exceed the transferrin carrying capacity, and as a result, the metal is complexed with low molecular-weight molecules, constituting the so-called non-transferrin bound iron (NTBI). NTBI can be taken up by hepatocytes, astrocytes and T lymphocytes. Importantly, in some circumstances, astrocytes and lymphocytes are capable of acquiring iron in the ferric form, suggesting the existence of a selective NTBI carrier [13,14]. It is worthy of note that iron in the form of NTBI has a much higher toxic potential [15].

In quantitative terms, the red blood cell (RBC) formation is the major consumer of iron in the body [10]. An efficient recycling system ensures that the iron resulting from the degradation of aged or damaged erythrocytes, degraded by the liver and spleen macrophages, is made available for all the body needs. During erythrophagocytosis, RBCs are contained inside a phagolysosome, and upon hemoglobin degradation, heme is transported to the cytoplasm by the protein heme-responsive gene, the homolog 1(HRG-1) [16]. In the cytoplasm, heme is either exported through the heme exporter, the feline leukemia virus subgroup C cellular receptor (FLVCR), [17] or catabolized into CO, biliverdin, and iron by HO-1 [18,19]. The regulation of heme catabolism and its export after erythrophagocytosis is important not only due to the toxicity of free heme but also because heme can be used by pathogens as a source of iron. In this context, the serum protein hemopexin plays an important protective role [20,21,22]. Hemopexin is responsible for heme-binding in the serum, protecting the cells from its nefarious effects, and for the subsequent transport of heme to the sites of iron recycling.

The iron released from the heme degradation is either stored intracellularly in ferritin or exported from the cell by FPN1, which is up-regulated during erythrophagocytosis [23,24]. FPN1 expression at the cell membrane is regulated by hepcidin. Hepcidin is a small peptide (coded by the Hamp1 gene) produced mainly in the liver, the expression of which is regulated by several stimuli, including tissue iron levels, anemia, hypoxia and inflammatory cytokines [25].

Macrophages are key players in both iron homeostasis and response to infections. On one hand, macrophages are responsible for iron recycling and storage. On the other hand, they play fundamental tasks in the immune response, by secreting different types of cytokines, directly killing phagocytosed microbes or serving as the host cell for others. It was proposed that specific stimuli act on macrophages, polarizing their activation towards M1 or M2 phenotypes [26]. Interferon gamma (IFNG), lipopolysaccharide (LPS), Tumor Necrosis Factor alpha (TNF) and Granulocyte-Macrophage Colony-Stimulating Factor (GM-CSF) induce macrophage polarization towards an M1 phenotype, with the production of pro-inflammatory cytokines and effector molecules, which mediate, for instance, the clearance of intracellular pathogens. On the flip-side, cytokines like IL-4 and IL-13, among others, activate macrophages towards an M2 phenotype, which facilitates tissue regeneration. This dichotomy is not always clear and intermediate polarization of phenotypes may occur. Regarding the iron metabolism, it has been described that M1 macrophages seem more prone to store iron whereas M2 macrophages seem keener in exporting iron [27]. On the other hand, high iron levels induce an M2 polarization, repressing the M1 phenotype [28]. These observations have important consequences in the regulation of iron metabolism during pathological situations, such as infections.

## 3. Different Pathogens, Different Impacts on the Iron Metabolism of the Host

First identified as a liver-derived antimicrobial peptide [29], hepcidin is now considered the main regulator of iron homeostasis in mammals. Hepcidin expression and release follow the pattern of acute phase proteins, being induced by pro-inflammatory cytokines, namely IL-6 [30,31]. The main effect of this peptide is to decrease the circulating iron levels, by binding and promoting the internalization and degradation of the only known cellular iron exporter, FPN1 [32]. The induction of hepcidin production by inflammatory mediators and microbial products, such as LPS, has been interpreted as a nutriprive host strategy to fight infectious agents. However, the real impact of hepcidin production and hepcidin-induced hypoferremia, in resistance to infection, has only recently been addressed, experimentally. It is now apparent that hepcidin-mediated protection is limited to certain types of pathogens [33,34].

Following the first studies showing a Toll-like receptor (TLR) 4-dependent induction of hepcidin by LPS, in vivo mouse studies showed increased levels of hepcidin during infections with *Salmonella* [35,36], *Pseudomonas aeruginosa*, group A *Streptococcus* [37], *Vibrio vulnificus* [38], and *Candida albicans* or Influenza A virus [39]. Additionally, in humans, several types of infections, including HIV, *Salmonella*, tuberculosis, sepsis, and malaria, have been reported to be accompanied by increased levels of serum hepcidin [40,41,42,43,44]. In marked contrast, the Hepatitis C virus inhibits hepcidin production in humans, which contributes to the pathology of this disease [45]. Interestingly, when infected with *Vibrio vulnificus*, *Y. enterocolitica* serotype O9 or *Klebsiella pneumoni*, a dramatic increase in pathogen growth and host mortality occurred in mice that were genetically deficient in hepcidin production [38,46,47]. Overall, data from these experiments showed that hepcidin decreased the levels of circulating iron and especially NTBI, which is critical to avoid the proliferation of highly siderophilic bacteria, such as *V. vulnificus* and particular strains of *Y. enterocolitica*, in the blood. In the case of the animal model of pneumonia caused by *Klebsiella pneumonia*, hepcidin mostly exerted a protective role also through an inhibition of the bacterial dissemination to the blood [46]. The host-protective role of hepcidin in these particular cases was further demonstrated by the significant therapeutic effect of hepcidin mimics [38,46,47]. Hepcidin administration or overexpression was also protective against the hepatic stage of *Plasmodium* infection, and may be implicated in the natural resistance to hepatic infections seen in the blood-stage carrying individuals [48].

In contrast, studies in *Hamp1*-knock-out (KO) mice showed that the lack of hepcidin had no effect on host resistance against *Yersinia enterocolitica* serotype O8, *Staphylococcus aureus* or *Mycobacterium tuberculosis* [47,49], presumably because these pathogens do not depend on an abundance of the serum NTBI, for their proliferation.

Another interesting observation made in the *Hamp1*-KO model was that LPS injection induces a significant degree of hypoferremia by hepcidin-independent mechanisms [50]. One such mechanism is the decrease in the transcription of the *Fpn1* gene, with a subsequent decrease in cell iron export. In fact, TLR4, but not TLR2 ligands were shown to induce hepcidin expression in myeloid cells, but both caused a down-modulation of FPN1 expression in vitro [51]. In vivo, different TLR agonists induced hypoferremia without a concomitant increase in the hepcidin expression but were accompanied by *Fpn1* down-regulation [50,52]. The same effect was seen in mice during infections by *Listeria monocytogenes*, a Gram-positive bacterium [35].

Most infections are thus accompanied by hypoferremia, which may result from the action of hepcidin and/or FPN1 down-modulation. This hypoferremia is concomitant with iron accumulation in the macrophages, which may favor the growth of intramacrophagic pathogens. This possible dilemma has been extensively investigated in *Salmonella enterica* serovar Typhimurium, with conflicting results.

In vitro studies with cultured macrophages showed that overexpression of FPN1 increases iron export and decreases the intracellular growth of *Salmonella* [53,54]. Furthermore, the treatment of these cells with hepcidin increased the growth of the bacteria [54,55]. It was also found that infection with *Salmonella* naturally induces FPN1 overexpression in macrophages, by an inducible nitric oxide synthase and nuclear factor erythroid 2-related factor 2 (Nrf2)-dependent pathway, resulting in an increase of iron export [53]. FPN1 expression inversely correlates with the intracellular growth of *Salmonella* and also of other intracellular bacteria, such as *Legionella* and *Chlamydia* [53,56].

In vivo experimental infections with *S*. Typhimurium, either by the intravenous or the oral route, consistently resulted in an increased expression of hepcidin in the liver and spleen, hypoferremia, and mild anemia [35,53,57]. However, contradictory results were reported with respect to the impact of *Salmonella* infections on FPN1 expression in the liver and spleen, as well as on the tissue iron levels, in these organs [35,53,57,58]. Interestingly, one study found that pharmacological inhibition of hepcidin production had a protective effect, inducing lower bacterial burden and decreased mortality by *Salmonella* infections [57,59], while in another study no differences were seen in bacterial growth between wild-type (WT) and *Hamp1*-KO mice [60]. Further studies are needed in order to explain these discrepancies, which may result from the different infection routes (intravenous, intraperitoneal or oral). The levels of hepcidin produced, and the related consequences for the kinetics of the iron dysregulation also need to be investigated. So far, the emerging picture is that although hepcidin is produced during *Salmonella* infection, it has no host-protective role, being even a possible disease promoter.

Other paradigmatic intra-macrophagic infections are those caused by mycobacteria. Despite extensive evidence of a link between host iron status and the severity of disease, in this particular type of infections, hepcidin does not seem to play a critical role in the host-pathogen interaction. Mice infected with *Mycobacterium avium* did not exhibit any significant alteration in hepcidin expression in the liver, while in animals, infected with *M. tuberculosis*, hepcidin expression was repressed at later time-points [49,61]. Moreover, hepcidin deficiency had no impact on susceptibility to *M. tuberculosis* [49]. In both experimental models of infection, with *M. avium* and *M. tuberculosis*, FPN1 expression in the liver was up-regulated [49,61] This up-regulation of FPN1 is probably related to expression in hepatocytes, rather than macrophages, since mice infected with *M. avium* exhibit iron accumulation inside infected macrophages, in the liver granulomas [62].

Another consequence of mycobacteria infection and iron accumulation inside macrophages is the induction of H-ferritin. Infection with *M. avium* or *M. bovis* BCG increased the expression of H-ferritin, upon activation of TLR2 in macrophages, in an Iron Regulatory Proteins (IRP)- independent manner [51,63]. Ferritins are iron storage proteins composed of a combination of 24 subunits of the H or L types. Ferritins are mainly thought of as intracellular iron storage proteins. H-ferritin has an oxidase activity, promoting the oxidation of Fe (II) to Fe (III), without which iron cannot be incorporated inside the ferritin cages. L-Ferritin facilitates the nucleation and mineralization of the iron core [64]. The expression of both the H-ferritin and L-ferritin genes is regulated post-transcriptionally by the IRPs. However, H-ferritin transcription is also responsive to inflammatory stimuli, including TNF and IL-2 [65,66,67]. Consequently, one of the iron-related hallmarks of the inflammatory response is hyperferritinemia (rise in the serum levels of ferritin) [64,68,69]. Serum ferritin levels are higher, for example, in the plasma of humans infected with *Plasmodium vivax* in comparison with non-infected individuals [70]. Serum ferritin was shown to be mostly composed of L-subunits with a few H-subunits, to have a low iron content, and to be derived from macrophages through a non-classical secretory pathway [71]. The exact role of the serum ferritin, and the mechanism triggering its release into circulation, during inflammatory conditions, are not known. Some controversy persists as to the role of ferritin in inflammation, with some authors defending that ferritin has an anti-inflammatory effect and others claiming that ferritin is pro-inflammatory [67,72].

Cell surface receptors for both subunits of ferritin have been described and are expressed in immune cells and in the liver [73,74,75]. It has been hypothesized that ferritin serves as an alternative source of iron but it may also be involved in the host’s strategy to overcome the potential cytotoxic effects of iron, during infections. H-ferritin has been recently reported to interfere with Hypoxia induced factor (HIF) 1α-mediated response to hypoxia [76]. In this regard, it is important to note that H-ferritin confers tolerance to malaria and sepsis through the decrease in tissue damage and independently from parasite load [70,77]. On the other hand, mice deficient in H-ferritin are more susceptible to *M. tuberculosis* infection, having higher bacterial loads and exacerbated inflammatory response [78]. These models highlight the importance of iron homeostasis, in the control of pathology, during an infection. It is thus evident that iron sequestration and re-distribution by the host has a key influence in the outcome of infectious processes, beyond its role in pathogen nutrient-deprivation.

In this context, another top contender in the host-pathogen interaction is heme. Heme is an important source of iron but is also highly toxic, and is an important cause of tissue damage. Heme transport and metabolism are, thus, handled very tightly within the host. It is increasingly evident that several infections lead to hemolysis, even if at a low level. This leads to increased levels of free hemoglobin and heme in the plasma, which in turn increases the production and release of hemopexin and haptoglobin, and also increases the expression of HO-1 in several tissues. Whether HO-1 activation is protective and/or detrimental for the host is a matter of discussion (reviewed in [79]). HO-1 expression in macrophages is induced upon an infection by mycobacteria and salmonella, both in vitro and in vivo. HO-1 induction has a protective role against these pathogens, being associated with a decrease in bacterial growth and also in oxidative stress-associated pathology [80,81,82]. Additionally, in vitro experiments showed that HO-1 is necessary for IFNG-induced autophagy and *M. tuberculosis* growth-arrest inside macrophages [83]. Moreover, the chemical induction of *Hmox1* (the gene which codes for HO-1) reduces the pathogen load of macrophages infected with *Trypanosoma cruzi* [84], and in vivo experiments with *Hmox1*-KO, or myeloid-specific *Hmox1*-deficient animals showed that these are more susceptible to sepsis caused by *E. coli* or *Listeria monocytogenes* [85]. Heme toxicity may be avoided by the binding of the molecule to proteins such as hemopexin and haptoglobin. IL-22 produced during an immune response to enteric pathogens, elicits the expression of hemopexin to scavenge the plasmatic heme [86]. This pathway reduces the growth of the bacteria, although it is not clear whether this results only from the removal of iron from the pathogens or also from the reduction of the cytotoxic effects of free plasma heme.

Contrasting results were obtained in different infection models. In mice, HO-1 induction was associated with immunomodulation and exacerbation of infection by *Fasciola hepatica* [87]. Experiments with chemical HO-1 inhibitors suggested that HO-1 was detrimental to the host infected with *M. tuberculosis* [88]. Interestingly, high levels of serum HO-1 were found in patients with active pulmonary tuberculosis, in contrast with latent *M. tuberculosis* carriers or healthy people [89,90]. Overall, these studies suggest that, although uniformly induced, HO-1 has specific effects, depending on the type and localization of the invading pathogen. In humans, polymorphisms in the promoter of *Hmox1*, associated with higher expression of the enzyme, correlate with a more severe presentation of malaria [91]. Murine models of malaria have shown that the higher expression of HO-1 correlated with higher hepatic parasite loads due to an attenuated inflammatory response, promoting the establishment of infections [92]. However, during the cerebral stage of malaria, induction of HO-1 does not alter parasitemia but prevents the tissue damage associated with the disease, maintaining the integrity of the blood-brain barrier and that of the brain microvasculature, and preventing neuroinflammation [93,94].

The intricate relationship between the HO-1 and infections gets another level of complexity in situations of co-infections. Malaria and other hemolytic disorders are often associated with non-typhoid *Salmonella* septicemia. It is now understood that during malaria-induced progressive hemolysis, the free heme resulting from the bursting erythrocytes is catabolized by the HO-1, releasing CO, biliverdin, and iron, thus reducing the ROS production by the phagocytes, and facilitate the bacterial replication. Therefore, in the context of co-infections, HO-1 mediates a protective response against malaria but impairs the immune response to non-typhoid *Salmonella* infections [95,96].

## 4. Nutritional Immunity and Infection-Induced Anemia

Infectious diseases, especially those with a chronic evolution, are often accompanied by anemia. For a long time, anemia of infection and inflammation was considered to be a side effect of nutritional immunity. According to this view, by lowering the levels of circulating iron in order to limit pathogen growth, the host makes iron unavailable for the formation of new red blood cells. Anemia of inflammation has thus been linked to hepcidin-mediated iron redistribution, lower levels of circulating iron and increased iron storage in tissues [97]. Experimental models of anemia of inflammation brought on by turpentine-induced sterile abscesses, caused systemic hypoferremia at the early stages of inflammation but it resolved at the later stages. However, anemia persisted due to inefficient development of erythrocyte maturation in the bone marrow [98]. Mice treated with heat-killed *Brucella abortus* developed hypoferremia at early stages with subsequent development of anemia [36]. Despite these observations linking hypoferremia to the development of anemia, the causal relationships between hepcidin, hypoferremia, and anemia in the context of specific infections have not been established and the mechanisms triggering anemia during infections are still not fully understood.

In some infections, systemic iron levels remain unchanged. For instance, *M. avium* infection induces anemia independently of hepcidin induction and hypoferremia [61]. The dysregulation of erythrocyte formation during infections may result from factors other than iron deficiency [99,100,101,102]. The inflammatory cytokine IFNG was implicated in the pathogenesis of several hematological disturbances, during infections, through its impact on the turnover of hematopoietic stem cells (HSC), either leading to their exhaustion or skewing their differentiation towards the myeloid lineage in detriment to other lineages, like the erythroid [99,100]. IFNG was also implicated in emergency erythropoiesis with reticulocytosis (i.e., increased circulating reticulocytes), in response to infections by *Plasmodium* [103]. Other cytokines, like IL-6 produced by the bone marrow stromal compartment, were shown to be involved in the impairment of erythropoiesis, during infections with *Toxoplasma gondii* [104].

Interestingly, hepcidin- or IL-6-deficient mice still developed anemia in response to heat-killed *Brucella abortus* injection. In this infection model, anemia had a multifactorial origin, including erythropoiesis impairment and reduced erythrocyte lifespan [36,105]. In this context, it is important to note that an increasing number of reports associate infectious diseases with the appearance of hemophagocytic macrophages. Hemophagocytes are myeloid cells with increased avidity to engulf erythrocytes and, in some instances, leukocytes. These cells are frequently observed during infections by pathogens, such as *Salmonella enterica*, *Brucella abortus*, Epstein-Barr virus and *M. tuberculosis* [106,107,108,109]. *S*. Typhimurium infections were shown to result in anemia and lead to the development of hemophagocytic macrophages, related to IFNG and IL-12-induced iron efflux from tissues [58]. The mechanisms activating the macrophage to engulf erythrocytes are not fully described, but there is some evidence suggesting that pro-inflammatory cytokines produced by the immune response to the ongoing infections, such as IFNG and TNF, stimulate macrophages to phagocytize erythrocytes, which in turn causes the development of anemia and other hematologic disorders [110,111]. In the absence of infections, the prolonged infusion of IFNG led to the appearance of hemophagocytic macrophages and the subsequent development of anemia [112,113]. Hemophagocytic macrophages that appeared in response to infection, had markers of anti-inflammatory M2 phenotype [114]. Genetic mutations may predispose patients to the development of hemophagocytes in response to infections [107,115].

The observation that during the inflammatory response, anemia may occur due to increased degradation of erythrocytes rather than decreased formation, represents an alternative view of anemia etiology and has important implications for the clinical management of infections and infection-induced anemia.

Increased erythrocyte degradation may result from different triggering events depending on the infectious agent. Two recent reports have demonstrated how the immune response itself may induce anemia, by the production of auto-antibodies against erythrocyte intrinsic proteins, during malaria [116]. The deposition of these auto-antibodies—purified from anemic malaria patients—on the surface of non-infected erythrocytes, altered the dynamic fluidity of the erythrocytes and led to their phagocytosis [117]. Type I interferons may also induce the exposure of phosphatidylserine (PS) on the surface of erythrocytes and the expression of PS receptors on myeloid cells, triggering erythrophagocytosis upon infection with lymphocytic choriomeningitis virus (LCMV) [118]. Along with cytokines, activation of TLR4 also stimulates hemophagocytosis [119]. On the other side, the pathogen may also have an active role in inducing alterations in erythrocytes, to cause their phagocytosis. *Trypanosoma vivax* produces and secretes enzymes that desialylate the host RBC, causing their erythrophagocytosis [120]. *S. Typhimurium* resides within macrophages containing phagocytized erythrocytes, which may benefit the acquisition of iron by the pathogen [121].

The engulfment of erythrocytes potentially increases the release of free heme, which as we discussed before, is an important player for the host and for the pathogen. Heme itself has the capacity to induce the differentiation of macrophages into an iron-recycling phenotype through the induction of the gene SpiC, degradation of the transcription factor BACH1, and Nrf2-mediated induction of HO-1, the high expression of which is a characteristic of hemophagocytic macrophages during sepsis [122,123]. Heme released during erythrocyte engulfment may be detrimental for the host response, besides providing iron for the pathogen, as it inhibits phagocytosis and the migration of phagocytes, due to a disruption of the cytoskeletal dynamics [85]. It is possible that the host developed a strategy to overcome these detrimental effects by the induction of ferroptosis in macrophages engaged in hemophagocytosis [124]. Ferroptosis is a regulated form of cell death due to the iron-dependent accumulation of lethal levels of lipid hydroperoxides [125]. Upon the death of ferroptotic macrophages, circulating and bone-marrow-derived monocytes are recruited to the tissues, where they differentiate into FPN1-expressing iron-recycling macrophages [126]. The development of this population of macrophages during situations of heavy hemophagocytosis is dependent on the growth factor, Csf1, and the transcription factor, Nrf2 [126]. Together, these observations suggest that iron induces important changes in the immune populations during the response to infection, implying that nutritional and immune responses are deeply interlinked and have to modulate each other, in order to maintain the host homeostasis and the capacity to fight the ongoing infection. The understanding of whether hemophagocytosis benefits the host immune response or the pathogen is important for the design of adjuvant therapies to treat infection and the associated anemia.

## 5. Targets for Pharmacological Intervention

In the context of infection, iron metabolism is an important therapeutic target for several reasons: (1) iron is needed by the pathogen; (2) iron dysregulation may lead to anemia; (3) iron may contribute to the pathology. A deeper understanding of the impact of infection on the host’s iron metabolism is thus needed in order to allow the planning of efficient therapeutic interventions. Some of the most recent strategies proposed to target host iron metabolism during infections, are summarized in Table 1.

A wide range of chelators (molecules with high affinity for iron) have been extensively designed and synthesized in the search for the treatment of diseases: from cardiac to neurodegenerative disorders and cancer [127,128]. As pathogens deeply need iron to survive, the introduction of chelators seems a plausible strategy to treat infections and overcome the increasing resistance developed by pathogens, to the available drugs. In vitro, the potential use of hexadentate iron chelators was shown to control mycobacteria growth [129]. The action of these molecules was improved by the conjugation of rhodamine fluorophores [130]. Iron chelators were shown to increase the efficacy of chemotherapeutic agents against *Candida albicans*, *Staphylococcus aureus* and polymicrobial sepsis [131,132,133]. Given the host’s need of iron for his own metabolic needs, one important concern related to iron chelation therapies is the development of adequate cell-targeting strategies that may guarantee pathogen’s iron depletion, without a concomitant iron deficiency in the host. Altogether, there is a huge potential for medical applications of iron chelators and the beneficial effects of different iron chelators were already shown in several experimental infections. However, chelators-based therapies have not been shown to be sufficiently effective in the treatment of infections in clinical trials [134,135].

One alternative to the administration of iron chelators is to block pathogen’s iron acquisition pathways. Lipocalin (Lcn)-1 and 2 are host proteins, with high affinity for iron-loaded microbial siderophores that restrict microbial growth by inhibiting iron uptake. In a mouse model of fungal corneal infection, the topical administration of Lcn-1or the inhibition of siderophore synthesis by the fungus, had a therapeutic effect with respect to iron starvation [136]. In a different approach to decrease the pathogen’s capacity to internalize iron, siderophores were successfully used as antigens for the development of a vaccine against uropathogenic *Escherichia coli* [137]. In the same line, a vaccine containing live *Yersinia pestis* was found to have a high potential therapeutic value, successfully limiting pathogen proliferation and the progression of the disease, by inducing the production of hemopexin and transferrin, with consequent iron sequestration [138]. The modulation of hepcidin function during infections is another potential target to control pathogen growth, as suggested by the observation that *Plasmodium* erythrocytic stages inhibit liver-stage superinfection, through hepcidin induction [139]. The use of minihepcidins, synthetic hepcidin agonists, are known to bind ferroportin and induce its internalization, was initially studied in iron overload disorders, such as hemochromatosis [140]. The administration of hepcidin mimetics was then tested in experimental models of infection and shown to clearly protect from *Vibrio* and *Yersinia*-induced mortality, by reducing bacterial loads. These hepcidin mimetics were shown to be effective not only in preventing but also at treating infections [38,46]. In the case of *Salmonella* infections, a therapeutic effect was seen with the pharmacological inhibition of hepcidin production [57].

Regarding the correction of anemia associated with infections, it is important to distinguish the cases involving hepcidin activity from those that do not. Several inhibitors of hepcidin production have been successfully tested to correct anemia of inflammation [141], but their usefulness in the context of infection awaits confirmation.

The above-mentioned examples show that pharmacological approaches to alter iron homeostasis and iron trafficking, during infections, may make an important contribution to the anti-infective therapeutic armamentarium. However, it should be kept in mind that different pathogens have different strategies to acquire iron in the host and the same intervention may have positive results in one case and negative or neutral consequences in others.

## 6. Conclusions

Infections lead to important alterations in the host’s iron metabolism, including iron redistribution within the tissues. Different pathogens have different impacts, some of which favor pathogen growth while others protect the host. We are still far from having a comprehensive picture of the pathways involved. Therefore, it is imperative that basic research work continues to be developed in order to gain a deeper understanding of the specific changes occurring in host iron homeostasis in response to each specific pathogen.

## Figures and Tables

**Table 1:** Iron-targeted strategies to fight infection.

Approach	Mechanism	Reference
Iron chelators	Direct reduction of available iron	[129,130,131,132,133,134]
Vaccines	Inhibition of iron uptake	[137,138,139]
Hepcidin agonists	Reduction of NTBI	[38,47]
Hepcidin inhibitors	Reduction of hepcidin production	[57]
